# The Novel Bone Alkaline Phosphatase Isoform B1x Is Associated with Improved 5-Year Survival in Chronic Kidney Disease

**DOI:** 10.3390/nu13124402

**Published:** 2021-12-09

**Authors:** Mathias Haarhaus, Anders Fernström, Abdul Rashid Qureshi, Per Magnusson

**Affiliations:** 1 Department of Clinical Chemistry, and Department of Biomedical and Clinical Sciences, Linköping University, SE-581 85 Linköping, Sweden; Per.Magnusson@regionostergotland.se; 2 Division of Renal Medicine and Baxter Novum, Department of Clinical Science, Intervention and Technology, Karolinska Institutet, Karolinska University Hospital, SE-141 86 Stockholm, Sweden; tony.qureshi@ki.se; 3 Diaverum Sweden AB, Hemvärnsgatan 9, SE-171 54 Solna, Sweden; 4 Department of Nephrology, and Department of Health, Medicine and Caring Sciences, Linköping University, SE-581 85 Linköping, Sweden; anders.fernstrom@regionostergotland.se

**Keywords:** survival, cardiovascular disease, arterial stiffness, vascular calcification, bone alkaline phosphatase, chronic kidney disease, dialysis

## Abstract

Circulating alkaline phosphatase (ALP) is an independent cardiovascular risk marker. Serum bone ALP (BALP) isoforms indicate bone turnover and comprise approximately 50% of total circulating ALP. In chronic kidney disease (CKD), mortality is highest in patients with increased ALP and BALP and low bone turnover. However, not all low bone turnover states are associated with increased mortality. Chronic inflammation and oxidative stress, features of protein energy wasting syndrome, induce cardiovascular BALP activity and fibro-calcification, while bone turnover is suppressed. Circulating BALP isoform B1x is associated with low ALP and low bone turnover and has been exclusively detected in CKD. We investigated the association of serum B1x with survival, abdominal aortic calcification (AAC) score, and aortic pulse wave velocity (PWV) in CKD. Serum ALP, BALP isoforms, parathyroid hormone (PTH), PWV, and AAC were measured repeatedly over 2 years in 68 prevalent dialysis patients. Mortality was assessed after 5 years. B1x was detected in 53 patients. A competing risk analysis revealed an association of B1x with improved 5-year survival; whereas, baseline PWV, but not AAC score, predicted mortality. However, PWV improved in 26 patients (53%), and B1x was associated with variation of PWV over time (*p* = 0.03). Patients with B1x had lower PTH and total ALP, suggesting an association with lower bone turnover. In conclusion, B1x is associated with time-varying PWV, lower circulating ALP, and improved survival in CKD, and thus may be an indicator of a reduced cardiovascular risk profile among patients with low bone turnover.

## 1. Introduction

Cardiovascular (CV) complications are the main reason for the high morbidity and mortality in patients with chronic kidney disease (CKD) on maintenance dialysis [1]. Profound evidence has accumulated for an underlying, complex interaction between bone and the vasculature [2]. Disturbances of bone turnover, especially low bone turnover, have been associated with CV complications in CKD. However, recent evidence suggests that low bone turnover, per se, may not have a detrimental effect on CV risk. Instead, underlying mechanisms, e.g., malnutrition and chronic inflammation, seem to determine the association of low bone turnover with negative outcomes in CKD [3].

Serum total alkaline phosphatase (ALP) associates with all-cause and CV mortality in different CKD populations and in the general population [4]. Increased bone alkaline phosphatase (BALP) may even be a better predictor of short- and long-term mortality than total ALP, especially in combination with low parathyroid hormone (PTH); indicative of an extra-skeletal origin of ALP or BALP in the setting of low bone turnover [5,6]. Vascular stiffening and accelerated vascular calcification are common in CKD and are associated with increased serum total ALP activity and increased mortality [7,8]. Chronic inflammation and oxidative stress, which are features of protein energy wasting syndrome, induce vascular ALP and fibro-calcification in cardiovascular tissue [9], and clinical indicators of malnutrition are associated with increased circulating ALP [10]. The process of vascular calcification involves the transdifferentiation of vascular smooth muscle cells (VSMC) to an osteoblast-like phenotype with increased expression of BALP [11]. Evidence has accumulated suggesting BALP as a key player, and possible therapeutic target, in skeletal and CV pathologies associated with CKD [3,4,12,13].

Four different BALP isoforms (B/I, B1x, B1, and B2) have been identified in bone tissue [14,15], VSMC [11], and serum [15,16,17] that share the same protein structure, but differ in post-translational glycosylation [18]. Biochemical and functional differences between these isoforms modulate their mineralization potential and could, thus, impact differently on cardiovascular- and bone-related outcomes in CKD [19,20,21]. The release of BALP into circulation is an active process [19,22]. It is of clinical importance that the BALP and liver ALP isoforms comprise approximately 50% each of the total circulating ALP activity, with small amounts (<1–10%) of intestinal ALP [9,23]. The BALP isoforms B/I, B1, and B2 are present in serum from healthy individuals and can be elevated, to various extents, in patients with different metabolic bone diseases [12]. Serum B1x is exclusively detected in some dialysis and pre-dialysis patients and is associated with lower activities of total ALP and the other BALP isoforms [16,17,24]. In dialysis patients, serum B1x has been identified as a marker of low bone turnover [25].

The aim of the current study was to investigate the relationship of the BALP isoforms (and more specifically, the B1x isoform), vascular stiffness (determined by aortic pulse wave velocity (PWV)), and abdominal aortic calcification (AAC) score with long-term survival, evaluated after 5 years, in a cohort of Swedish dialysis patients.

## 2. Materials and Methods

### 2.1. Patients

Patients participating in this multicenter prospective cohort study were recruited from the CORD (Calcification Outcome in Renal Disease) study cohort. The current study was a pre-defined sub-study, prospectively including patients from four Swedish dialysis centers (Karlstad Central Hospital, Linköping University Hospital, Malmö University Hospital, and Uppsala University Hospital). The sub-study was approved by the research ethics committee of Uppsala University, Sweden (2004: M-326). The inclusion process for the CORD study has been reported previously [7,26]. In brief, all patients 18 years of age or older and treated by peritoneal dialysis or maintenance hemodialysis for ≥3 months were screened. Patient enrollment followed selection by a computer algorithm, which guaranteed the inclusion of a representative sample of at least 20% of all screened patients per center, with regard to gender, age, smoking status, diabetes, and time on dialysis. Exclusion criteria were conditions making reliable measurements of PWV technically impossible (cardiac arrhythmias, amputations, severe peripheral vascular lesions) and a life expectancy of <6 months, according to the treating physician. No additional inclusion or exclusion criteria were applied in the current study. Patients were followed from the inclusion date until renal transplantation, death, or completing 5 years of follow-up. Patients were censored at 5 years or at the time of kidney transplantation, whichever came first. Events were adjudicated by the principal investigator of the CORD study at each participating center, who extracted the information from the patients’ charts.

### 2.2. Biochemical Determinations

Blood samples were retrieved at the time of PWV measurements. In hemodialysis patients, samples were retrieved prior to start of a dialysis session. The serum levels for calcium, phosphorus, and intact PTH, were determined by accredited routine clinical laboratory methods and retrieved from the patient records. Serum total ALP activity was measured using a kinetic assay in a 96-well microtitre plate format. In brief, a total volume of 300 µL solution was added per well, containing 1.0 M diethanolamine buffer at pH 9.8, 1.0 mM MgCl_2_, and 10 mM p-nitrophenylphosphate. The time-dependent increase in absorbance at 405 nm (reflecting p-nitrophenol production) was determined on a Multiscan Spectrum microplate reader (Thermo Electron Corp., Vantaa, Finland).

The BALP isoforms B/I, B1x, B1, and B2 were determined by a previously described high-performance liquid chromatography method [27,28]. In brief, the BALP isoforms were separated using a gradient of 0.6 M sodium acetate on a weak anion-exchange column, SynChropak AX300 (250 × 4.6 mm I.D.) (Eprogen, Inc., Darien, IL, USA). The column effluent was mixed on-line with the substrate solution, 1.8 mM p-nitrophenylphosphate in a 0.25 M diethanolamine buffer at pH 10.1. The ensuing reaction was developed in a packed-bed post-column reactor at 37 °C, and the formed product (p-nitrophenol) was directed on-line through a detector set at 405 nm. The areas under each peak were integrated, and the total ALP activity was used to calculate the relative activity of each of the detected BALP isoforms. The relation between the enzymatic activity units kat and U is 1.0 μkat/L corresponds to 60 U/L. Detection limits for the BALP isoforms were: 0.01 µkat/L (B/I), 0.02 µkat/L (B1x and B1), and 0.03 µkat/L (B2). The intra- and inter-assay coefficients of variation were <5% and <6%, respectively, for each of the BALP isoforms. Serum intact fibroblast growth factor 23 (FGF23) was analyzed using a two-site monoclonal antibody-based enzyme-linked immunosorbent assay (Kainos Laboratories Inc., Tokyo, Japan). The detection limit for the FGF23 assay was 3 ng/L, and the intra- and inter-assay coefficients of variation were <5% and <6%, respectively.

### 2.3. PWV

Patients were examined in the supine position and before a hemodialysis session or after drainage of the abdominal cavity in peritoneal dialysis patients. All measurements were performed by trained research personnel. PWV was determined by sequential recording of electrocardiogram-gated femoral and carotid artery pulse waves using applanation tonometry (SphygmoCor version 7, AtCor Medical, Sydney, Australia). The corrected path length was calculated by subtracting the distance between the sternal notch and carotid recording site from the distance between the sternal notch and femoral site. PWV was determined by dividing the corrected path length by the transit time (m/s). Measurements were performed at baseline, and after 1 and 2 years of follow-up.

### 2.4. AAC Score

Calcification scores were determined at baseline and after a follow-up period of 2 years, using a semi-quantitative analysis of a plain lateral lumbar radiograph including the aorta (AAC score), as previously described and validated [29]. All radiographs were scored by a single investigator, who was blinded to patient data.

### 2.5. Statistical Analysis

Data were analyzed using IBM SPSS Statistics 21.0.0 (IBM SPSS Inc., Chicago, IL) and SAS version 9.4 (SAS Campus Drive, Cary, NC). A critical significance level of 0.05 was chosen. Due to the skewness of some variables, paired comparisons were performed using the Mann–Whitney U test for continuous variables and χ^2^ statistics for discrete variables. Nonparametric correlations were calculated using Kendall’s tau rank correlation coefficient. Mixed model regression analysis was used to identify determinants of variation of PWV and AAC score over time, with visits, smoking status, BMI, FGF23, calcium × phosphate, PTH, ALP, and B1x as covariates. Predictors of 5-year mortality were identified by competing risk analysis. We used Fine and Gray models for competing-risk regression, with renal transplantation as a competing risk, to establish cumulative incidence curves [30,31,32]. These curves represent patients with the presence or absence of serum B1x, adjusting for sex and 1-SD increase of age and PWV. Risk estimates for patients with the presence of serum B1x expressed as sub-hazard ratios (sHR), and patients with the absence of serum B1x were used as a reference.

Due to the lack of previous studies linking BALP isoforms to mortality, no formal power analysis was performed. Sample size was based on feasibility, with the method of BALP isoform determination as the limiting factor. Therefore, only Swedish centers participating in the CORD study were asked to participate in the current sub-study.

## 3. Results

### 3.1. Patients

**Figure 1:**
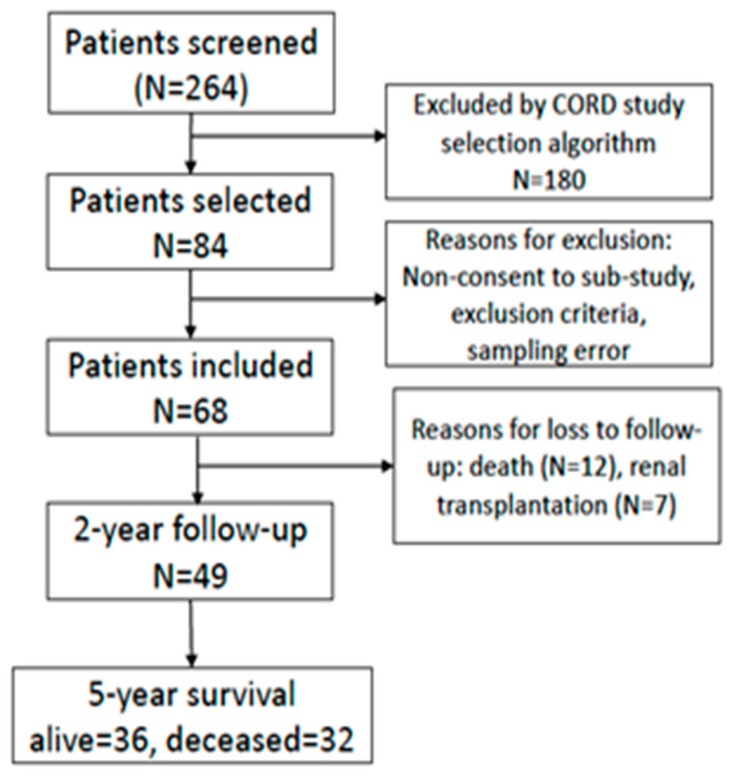
Flow chart of prospective patient enrolment and study procedure.

A total of 84 out of 264 patients, screened at the centers participating in this sub-study, were included in the CORD study [7,26]. Of these, 68 patients were included in the current study (Figure 1). Baseline characteristics of the included patients are summarized in Table 1.

### 3.2. BALP Isoforms

**Figure 2:**
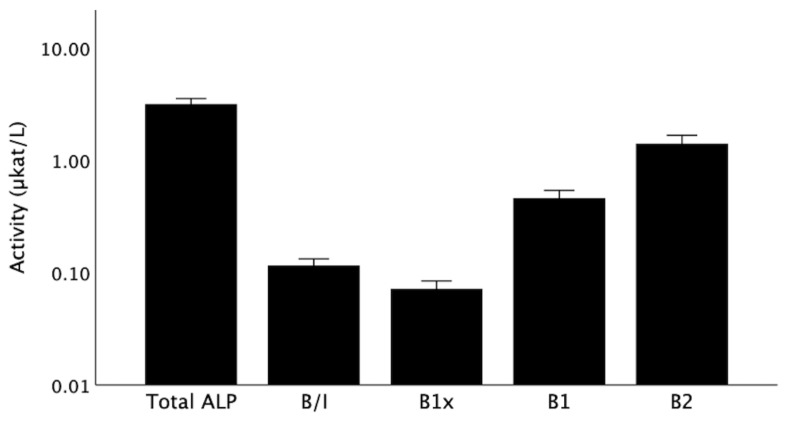
Total ALP and BALP isoforms at baseline. Data are shown as medians with error bars indicating a 95% confidence interval. ALP, alkaline phosphatase; B/I, B1x, B1, B2, isoforms of bone alkaline phosphatase.

The baseline serum activities for total ALP and the BALP isoforms (B/I, B1x, B1, and B2) are shown in Figure 2. The isoforms correlated positively with each other and with total ALP, with the exception of B1x, which demonstrated a weaker correlation, which was only significant for B/I and B1 (Table 2). Isoform B1, which is predominantly of cortical bone origin, was more strongly correlated with parathyroid hormone (PTH), in comparison with the other isoforms (Table 2).

Fifty-three patients (78%) had detectable serum B1x activities. Baseline characteristics of patients with and without B1x are listed in Table 3. Patients with B1x were older and had lower levels of total ALP, all other BALP isoforms, and PTH, while there was a tendency towards higher albumin.

### 3.3. PWV and AAC Score

**Figure 3:**
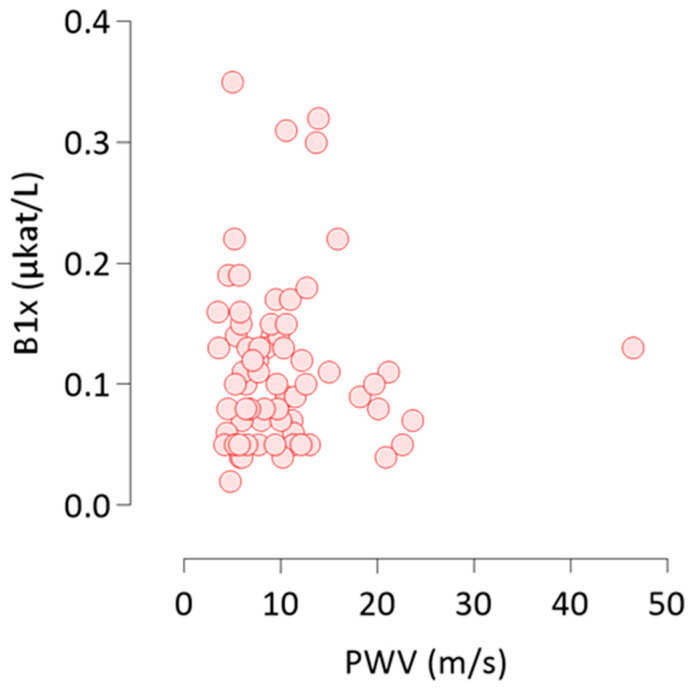
Non-parametric Kendall’s tau rank correlation of B1x with PWV at baseline. B1x, bone alkaline phosphatase isoform; PWV, pulse wave velocity.

All 68 patients recorded baseline measurements of PWV and AAC score. Both, AAC score (Kendall’s tau = 0.44, *p* < 0.0001) and PWV (Kendall’s tau = 0.36, *p* < 0.0001) correlated strongly with age and with each other (Table 2). B1x correlated positively with PWV (Figure 3), but not with AAC score at baseline (Table 2). None of the other BALP isoforms, total ALP, or FGF23 correlated with PWV or AAC score.

At the end of the 2-year follow-up period, AAC score was measured in 46 patients and PWV in 49 patients. The AAC score increased in 30 patients (65%) and decreased in two patients (4%). PWV increased in 22 patients (45%) and decreased in 26 patients (53%) during follow-up. Patients, whose AAC score increased during the 2-year follow-up had shorter dialysis duration (median (minimum-maximum) 17.5 months (3–192 months) vs. 33 months (6–180 months), *p* = 0.04), compared with patients with stable or decreasing AAC score. Patients whose PWV decreased during the 2-year follow-up had lower median body mass index (BMI) at baseline (23.3 kg/m^2^ (18.2–33.0 kg/m^2^) vs. 27.7 kg/m^2^ (19.8–42.2 kg/m^2^), *p* = 0.003) and were treated with lower doses of calcium-based phosphate binders (750 mg elemental calcium daily (250–3000 mg) vs. 1500 mg (750–6000 mg), *p* = 0.001), compared with patients with stable or increasing PWV. All other baseline parameters were not significantly different between groups. The AAC score increased somewhat less in patients with decreasing PWV (1 (−3 to 10)) than in patients with stable or increasing PWV (2.5 (0 to 21)), but the difference did not reach significance (*p* = 0.07). In the mixed model analyses, variation of PWV was independently associated with the variation of B1x over time (Table 4). No associations of variation of B1x with the variation of AAC score over time were detected (data not shown).

### 3.4. Predictors of 5-Year Mortality

**Figure 4:**
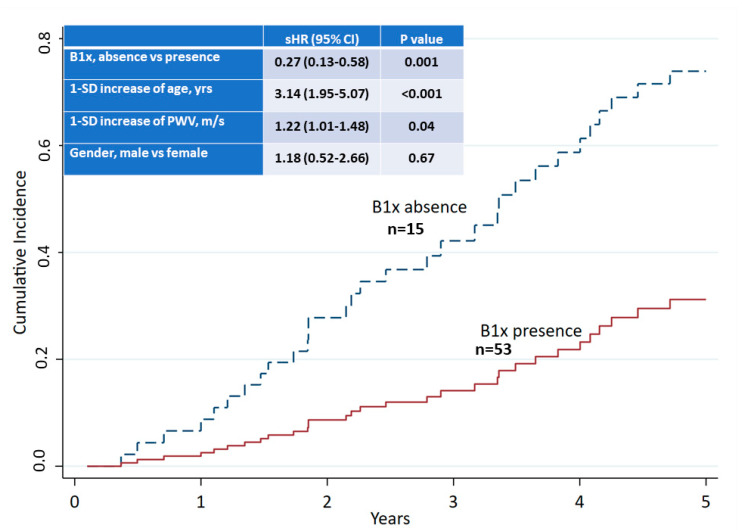
Competing risk analysis of 5-year all-cause mortality and presence or absence of B1x in serum at baseline.

During the follow-up to 5 years from baseline, 32 patients died, resulting in a 5-year mortality of 47%. At baseline, male sex, a history of CV disease, older age, lower albumin, and higher systolic blood pressure, pulse pressure, PWV, and AAC-score were associated with 5-year mortality (Table 5). A competing risk analysis demonstrated an independent association of the detection of B1x in serum with improved survival (Figure 4).

## 4. Discussion

This is the first study to demonstrate a relationship of the novel BALP isoform B1x with vascular stiffness and survival in CKD. The main findings were the association of serum B1x with baseline PWV, PWV variation over time, and with improved long-term survival. These findings are novel and hypothesis generating. Future research is warranted, to elucidate their clinical implications.

Observational studies have repeatedly demonstrated linear associations between ALP or BALP serum activities and mortality, as well as CV complications in CKD and non-CKD populations [4]. However, low BALP has also been suggested as a biomarker of low bone turnover, a form of renal osteodystrophy, which may be associated with increased mortality and CV complications. Recently, the concept that low bone turnover, per se, increases the risk for negative outcomes in CKD has been challenged [3]. While some systemic pathophysiologic processes associated with low bone turnover, e.g., malnutrition, chronic inflammation, or diabetes mellitus, are also frequently associated with increased mortality and CV complications [33,34], other forms of reduced bone turnover, e.g., as seen in treatment with antiresorptive agents, are not [35,36]. In some previous studies, correction for indicators of malnutrition and inflammation attenuated the observed association of low PTH states with mortality [3]. Drechsler et al. [4] demonstrated that dialysis patients with the lowest levels of PTH and BALP had the best short-term and long-term survival, whereas mortality was highest in patients with the highest BALP and lowest PTH, suggesting a role for BALP as a biomarker that can differentiate between low PTH states with divergent clinical outcomes. We recently confirmed similar associations for total ALP and PTH with mortality in peritoneal dialysis patients [6]. While experimental CKD seems to induce a very early expression of TNALP in rat vascular tissue, with concomitant calcification in areas of TNALP expression, these early changes are not related to circulating PTH levels [37]. On the other hand, PTH induction of vascular calcification seems to involve the RANKL/OPG system [38]. Thus, circulating PTH or stimulation of the PTH1R in VSMCs seems not to be directly related to increased TNALP or BALP isoform activities in vascular tissue. In the current study, patients with B1x demonstrated a tendency towards higher serum albumin concentrations. In addition, and consistent with previous studies [3], patients with circulating B1x had lower circulating activities of total ALP and BALP than patients without detectable serum B1x. We identified the presence of B1x in serum, rather than a specific cut-off level for B1x serum activity, as predictive of long-term survival. The presence of B1x in serum has previously been identified as a biomarker for low bone turnover in hemodialysis patients [22]. Based on the findings in the current study, we hypothesize that the detection of B1x in serum may indicate more favorable forms of low bone turnover.

While experimental VSMC calcification is associated with B1x isoform activity in the cell layer [8], in the current study, we could not establish an association between circulating B1x and aortic calcification. ALP is an ectoenzyme, i.e., a plasma membrane-bound enzyme, that has a catalytic activity outside of the cell [39]. ALP becomes located to specific areas of the cell membrane, which then are released through budding, to form matrix vesicles highly concentrated with ALP molecules with the capacity to mineralize [3]. It has been demonstrated that cultures of osteoblast-like cells release large amounts of ALP spontaneously into the cell medium, and that these amounts of ALP are substantially higher than detected in the cell layer [40,41]. However, the specific processes regulating this release of ALP and, more specifically, the BALP isoforms from tissues are not well described. In addition to a potential actively regulated contribution of tissue ALPs to circulating ALP activities, an active regulation of circulating ALP by serum neuraminidases has recently been suggested, with possible relevance for clinical outcomes [24]. Recent studies have also suggested ALP as a potential therapeutic target for the treatment and prevention of CV disease [4,9,13,42,43]. It is, therefore, of interest to further explore the specific roles of the BALP isoforms, and especially B1x, as biomarkers and potential therapeutic targets in CV disease.

In the current study, decreasing arterial stiffness was observed in more than 50% of patients who performed a follow-up PWV measurement, in spite of stable or increasing AAC scores. This decrease was associated with longer dialysis vintage, lower doses of calcium-containing phosphate binders, and lower BMI at baseline. Several longitudinal studies have determined the change of PWV in dialysis patients. Most studies reported a regression of PWV over time [44,45,46,47], whereas Utescu et al. [48] described an association of carotid–femoral PWV progression, with decreased carotid–brachial PWV, indicating possible differences between these vascular beds. It has been suggested that therapeutic interventions that can reduce ALP and bone turnover, e.g., treatment with cinacalcet or vitamin D and parathyroidectomy, have the potential to improve arterial stiffness [47,49,50]. Besides vascular calcification, endothelial dysfunction and CV fibrosis are additional modifiable mechanisms linking ALP to arterial stiffness [9], while pharmacologic lowering of ALP has recently been associated with improved CV outcome [13].

In accordance with the findings in the main CORD study, we found an association between PWV and AAC at baseline, but this association was not observed in the highly calcified patients who developed the CORD study’s combined endpoint of all-cause mortality or a first CV event. Verbeke et al. [7] speculated that extensive vascular calcification could cause an underestimation of PWV. An additional reason could be differences in the type of vascular calcification identified by these methods. Whereas intimal calcification predominates in the abdominal aorta [51], where the AAC score is determined, carotid-femoral PWV is also influenced by the thoracic aorta, where medial calcification predominates [52]. Calcifying VSMCs from the aortic arch are more prone to expressing ALP in comparison with VSMCs from the abdominal aorta [53]. Experimental studies have demonstrated a role for ALP in the propagation of tissue calcification [54,55], and we have previously described the putative greater importance of B/I and B1x, in comparison with B1 and B2, in this process [11]. Isoform B2 has the greatest potential to degrade the calcification inhibitor pyrophosphate [20] and also has the highest binding capacity to collagen I [21]. However, mechanisms of a possible active regulation of BALP isoforms in calcification-competent cells have not yet been described. It is too early to speculate whether the association of an increased activity of circulating ALP with mortality is mainly driven by systemic inflammation, a neutral or even positive bone balance, or the direct effects of cardiovascular calcification on the circulating BALP isoforms.

The association of vascular calcification with arterial stiffness grows weaker with age in individuals without CKD [56]. In CKD, increased arterial stiffness is associated with the vascular remodeling related to premature aging [57]. We found that the patients with detectable B1x were older than patients without B1x. However, the influence of vascular senescence on serum BALP isoform activities remains elusive.

The current study is the largest investigation that has been performed, comprising BALP isoforms in a cohort of CKD patients. The proportion of patients with detectable B1x in serum was somewhat higher than in previous studies [16,17,25]. This might be explained by changes in conventional treatment strategies for CKD–mineral and bone disorder, from the time of earlier studies to the time period of the current study, including the publication of the National Kidney Foundation Kidney Disease Outcomes Quality Initiative guidelines on bone and mineral metabolism [58] and the approval of cinacalcet by the Swedish Medical Products Agency, both of which could have influenced treatment strategies towards a more pronounced PTH suppression. The median PTH of patients with B1x was lower in the current study than in our previous study on BALP isoforms in CKD stage 5 on dialysis [16]. Differences in the size of the cohorts and the process of patient selection could be other explanations. Finally, possible changes in the use of phosphate binders and vitamin D between this and previous studies cannot be ruled out.

The strengths of the current study are its design as a prospective multicenter study of prevalent dialysis patients, a follow-up period of 5 years, and the use of a highly specific method for the detection and separation of BALP isoforms. However, the small number of patients is a major limitation, restricting the number of possible covariates in the multivariate analyses. In addition, the observational character of the study does not permit any causative deductions. Thus, the apparent discrepancy between a positive correlation of B1x with PWV at baseline and the association of B1x with improved 5-year survival, independent of PWV, on the other hand, cannot be explained by the current investigation. Larger studies are needed to confirm and elaborate the findings of this hypothesis-generating study. Further limitations are the diversity of the patients studied, with respect to dialysis vintage and medical treatment; the predominantly Caucasian origin of the participants; the possibility of a selection bias of the recruiting physician, in spite of the randomization process, which in fact would imply an underestimation of the actual risk in dialysis patients; and the large loss of patients during follow-up, which was mainly due to the high event rate.

In conclusion, we demonstrated that the novel BALP isoform B1x is associated with improved long-term survival in dialysis patients. In addition, B1x was associated with baseline aortic stiffness and variation of aortic stiffness over time in a Swedish population of prevalent dialysis patients. Further experimental and clinical studies are warranted, to elucidate the exact function of B1x in the processes linking bone and vascular pathologies and its possible role as a biomarker and potential future therapeutic target for the treatment and prevention of skeletal and CV complications in patients with CKD.

## Figures and Tables

**Table 1:** Baseline characteristics of all patients.

Characteristic	All Patients (N = 68)
Age (years)	68 (26–85)
Male	38 (56)
Smoking	8 (12)
BMI (kg/m^2^)	24.1 (15.6–42.2)
Sbp (mm Hg)	142 (81–200)
Dbp (mm Hg)	75 (30–100)
History of CV disease	33 (49)
Diabetes	19 (28)
Dialysis duration (months)	21 (3–192)
Hemodialysis VDRA Calcium carbonate Sevelamer	56 (82) 43 (63) 48 (71) 42 (62)
ALP (µkat/L)	2.9 (0.5–9.8)
B/I (µkat/L)	0.10 (0.02–0.35)
B1x (µkat/L)	0.08 (0–0.22)
B1 (µkat/L)	0.36 (0.07–2.09)
B2 (µkat/L)	1.13 (0.22–6.39)
PTH (pg/mL) FGF23 (pg/mL)	166 (7–1960) 10,426 (414–203,763)
Calcium (mg/dL)	9.5 (6.13–11.94)
Phosphorus (mg/dL)	5.1 (3.1–9.6)
PWV (m/s)	9.1 (3.5–46.5)
AAC score	12 (0–24)

Values are median (minimum–maximum) or n (%). AAC, abdominal aortic calcification; ALP, alkaline phosphatase; BALP, bone alkaline phosphatase; B/I, B1x, B1, B2, isoforms of bone alkaline phosphatase; BMI, body mass index; CV, cardiovascular; Dbp, diastolic blood pressure; FGF23, fibroblast growth factor 23; PTH, parathyroid hormone; PWV, pulse wave velocity; Sbp, systolic blood pressure; VDRA, vitamin D receptor agonists.

**Table 2:** Univariate correlation coefficients at baseline.

	FGF23	ALP	B/I	B1x	B1	B2	AAC Score	PWV
ALP	0.086							
B/I	0.069	0.499 ^c^						
B1x	0.114	0.125	0.277 ^b^					
B1	0.042	0.590 ^c^	0.657 ^c^	0.187 ^a^				
B2	0.079	0.768 ^c^	0.433 ^c^	0.133	0.484 ^c^			
AAC score	−0.015	−0.031	−0.006	0.021	−0.083	−0.006		
PWV	−0.085	−0.001	−0.004	0.233 ^b^	−0.064	0.030	0.329 ^c^	
PTH	0.119	0.252 ^b^	0.165	−0.053	0.302 ^c^	0.192 ^a^	−0.186 ^a^	−0.106

AAC, abdominal aortic calcification; ALP, alkaline phosphatase; BALP, bone alkaline phosphatase; B/I, B1x, B1, B2, isoforms of bone alkaline phosphatase; FGF23, fibroblast growth factor 23; PTH, parathyroid hormone; PWV, pulse wave velocity. ^a^: *p* < 0.05. ^b^: *p* < 0.01. ^c^: *p* < 0.001.

**Table 3:** Baseline characteristics of patients with or without B1x.

Characteristic	B1x Absent (N = 15)		B1x Present (N = 53)		*p*
Age (years)	59 (26–82)		68 (31–85)		0.049
Male	8	(53)	30	(57)	0.82
Smoking	0	(0)	8	(15)	0.11
BMI (kg/m^2^)	26.0 (19.4–38.3)		23.9 (15.6–42.2)		0.31
Sbp (mm Hg)	154 (81–200)		140 (100–200)		0.32
Dbp (mm Hg)	80 (50–90)		73 (30–100)		0.51
History of CV disease	5	(33)	28	(53)	0.18
Diabetes	5	(33)	14	(26)	0.60
Dialysis duration (months)	26 (6–156)		19 (3–152)		0.22
Hemodialysis VDRA Calcium carbonate Sevelamer	11 9 10 9	(73) (60) (67) (60)	45 34 38 23	(85) (64) (72) (43)	0.30 1.0 0.95 0.40
ALP (µkat/L)	4.1 (1.7–9.8)		2.7 (0.5–6.1)		0.008
B/I (µkat/L)	0.14 (0.06–0.35)		0.10 (0.02–0.32)		0.046
B1 (µkat/L)	0.63 (0.25–2.09)		0.33 (0.07–1.15)		0.001
B2 (µkat/L)	1.87 (0.57–6.39)		1.04 (0.22–4.07)		0.02
PTH (pg/mL)	390 (81–1960)		131 (7–1088)		0.003
FGF23 (pg/mL)	9389 (972–191,064)		10,907 (414–203,763)		0.79
Phosphorus (mg/dL)	5.3 (3.1–9.6)		5.0 (3.1–9.0)		0.51
Calcium (mg/dL) Albumin (g/L)	9.34 (8.42–10.58) 32 (26–40)		9.50 (6.13–11.94) 35 (21–44)		0.62 0.07
PWV (m/s)	6.0 (3.5–13.7)		9.6 (3.6–46.5)		0.048
AAC score	10 (0–24)		12 (0–24)		0.63

Values are median (minimum–maximum) or n (%). AAC, abdominal aortic calcification; ALP, alkaline phosphatase; BALP, bone alkaline phosphatase; B/I, B1x, B1, B2, isoforms of bone alkaline phosphatase; BMI, body mass index; CV, cardiovascular; Dbp, diastolic blood pressure; PTH, parathyroid hormone; FGF23, fibroblast growth factor 23; BMI, body mass index; PWV, pulse wave velocity; Sbp, systolic blood pressure; VDRA, vitamin D receptor agonists.

**Table 4:** Mixed model for the prediction of PWV variation over time.

Variable	Estimate	Standard Error	*p*-Value
Smoker vs. non-smoker	3.25	1.99	0.11
VISIT 2 versus baseline	−0.75	0.62	0.23
VISIT 3 versus baseline	−0.16	0.67	0.81
BMI (kg/m^2^)	−0.0089	0.11	0.94
FGF23 (pg/mL)	−0.00002	0.0000094	0.08
Calcium × phosphate product	−0.39	0.28	0.17
PTH (pg/mL)	0.00063	0.0011	0.58
Total ALP (µkat/L)	−0.11	0.19	0.56
B1x (µkat/L)	12.62	5.89	0.03

FGF23, fibroblast growth factor 23; VISIT 2, follow-up at 12 months; VISIT 3, follow-up at 24 months; BMI, body mass index; PTH, parathyroid hormone; ALP, alkaline phosphatase; B1x, isoform of bone alkaline phosphatase.

**Table 5:** Baseline characteristics of patients, depending on 5-year all-cause mortality.

Characteristic	Survivors (N = 36)		Deceased (N = 32)		*p*
Age (years)	58 (26–81)		74 (52–85)		<0.001
Male	16	(44)	22	(69)	0.43
Smoking	4	(13)		(13)	0.89
BMI (kg/m^2^)	24.1 (18.2–42.16)		24.1 (15.6–38.3)		0.59
Sbp (mm Hg)	136 (81–190)		150 (100–200)		0.03
Dbp (mm Hg)	80 (30–95)		73 (30–100)		0.31
History of CV disease	11	(31)	22	(69)	0.002
Diabetes	8	(23)	11	(34)	0.30
Dialysis duration (months)	17 (4–192)		28 (3–52)		0.35
Hemodialysis	27	(75)	29	(90)	0.09
ALP (µkat/L)	2.6 (0.5–9.8)		3.0 (1.4–6.1)		0.18
B/I (µkat/L)	0.10 (0.02–0.35)		0.10 (0.04–0.32)		0.71
B1x (µkat/L)	0.07 (0.0–0.15)		0.08 (0.0–0.22)		0.90
B1 (µkat/L)	0.35 (0.07–2.09)		0.36 (0.16–1.28)		0.57
B2 (µkat/L)	1.00 (0.22–6.39)		1.27 (0.31–4.07)		0.48
PTH (pg/mL)	203 (14–1960)		131 (7–1088)		0.91
FGF23 (pg/mL)	10,426 (414–203,763)		9598 (520–106,666)		0.88
Phosphorus (mg/dL)	1.6 (1.0–3.1)		1.7 (1.0–2.7)		0.78
Calcium (mg/dL) Albumin (g/L)	2.35 (1.53–2.98) 35 (21–44)		2.37 (2.07–2.93) 33 (26–40)		0.56 0.005
PWV (m/s)	6.6 (3.6–19.7)		9.8 (3.5–46.5)		0.007
AAC score	6 (0–23)		19 (0–24)		<0.001

Values are median (minimum–maximum) or n (%). AAC, abdominal aortic calcification; ALP, alkaline phosphatase; BALP, bone alkaline phosphatase; B/I, B1x, B1, B2, isoforms of bone alkaline phosphatase; BMI, body mass index; CV, cardiovascular; Dbp, diastolic blood pressure; PTH, parathyroid hormone; FGF23, fibroblast growth factor 23; PWV, pulse wave velocity; Sbp, systolic blood pressure.

## Data Availability

Data presented in this study are available on request from the corresponding author.
